# Hematological Profiles of Adults Coinfected With HIV and Malaria Receiving Highly Active Antiretroviral Therapy at Bonga Gebretsadik Shawo General Hospital, Southwest Ethiopia: A Comparative Cross-Sectional Study

**DOI:** 10.1155/ah/3894305

**Published:** 2025-08-21

**Authors:** Fikre Demango, Edosa Tadasa, Girum Tesfaye Kiya

**Affiliations:** ^1^Department of Medical Laboratory, Bonga Gebretsadik Shawo General Hospital, Bonga, Ethiopia; ^2^School of Medical Laboratory Sciences, Faculty of Health Sciences, Institute of Health, Jimma University, Jimma, Ethiopia

**Keywords:** hematological profiles, highly active antiretroviral therapy, HIV-malaria coinfection, parasite density, viral load

## Abstract

**Background:** Malaria and human immunodeficiency virus/acquired immunodeficiency syndrome (HIV/AIDS) are widely recognized infectious diseases that pose serious public health challenges in Sub-Saharan Africa and around the globe. A key factor contributing to the rise in human deaths related to malaria and HIV/AIDS is how these diseases can change the hematological parameters in people who are infected with both. Despite the significant effect of malaria and HIV/AIDS on hematological parameters, there are limited data regarding hematological profiles among malaria-HIV coinfected cases. Therefore, this study aimed to determine the hematological profiles of HIV-malaria-coinfected adults receiving highly active antiretroviral therapy at Bonga Gebretsadik Shawo General Hospital.

**Methods:** A hospital-based comparative cross-sectional study was conducted among 196 HIV-infected patients (98 HIV-infected and 98 HIV-malaria coinfected) at Bonga General Hospital from 13 June to 3 November 2022. Five milliliters of venous blood samples were collected to detect parasites, estimate parasite density, measure viral load, and perform a complete blood count. Sociodemographic data were collected using structured questionnaires. Data were analyzed using SPSS Version 25. Descriptive statistics, independent samples *t*-tests, and Spearman correlation tests were performed. A *p* value of < 0.05 was set as the cutoff for significance.

**Results:** The study included 196 adults living with HIV. Statistical differences were observed in the mean ± SD values of red blood cells, hemoglobin, and hematocrit (*p* < 0.05) between HIV-infected and HIV-malaria coinfected study participants. In a total of study participants, significant negative correlations were found between viral load and total white blood cell count, neutrophils, lymphocytes, eosinophils, red blood cells, hemoglobin, hematocrit, mean cell volume, and platelet count. Anemia, leukopenia, and thrombocytopenia were present in 88 (44.9%), 77 (39.3%), and 50 (25.5%) of the 196 participants, respectively. In the HIV-malaria-coinfected group, there was a negative correlation between parasite density and red blood cell count, hemoglobin, hematocrit, and platelets. The prevalence of anemia, leukopenia, and thrombocytopenia among malaria and HIV-coinfected study participants was 60 (61.2%), 43 (43.88%), and 30 (30.6%), respectively. A statistically significant difference (*p* < 0.001) was observed in the prevalence of anemia between the two groups.

**Conclusion and Recommendations:** The prevalence of anemia was significantly higher in HIV-malaria-coinfected participants than HIV monoinfected paricipants. Mean values of hematological profiles were significantly different in the two groups. Further studies with a larger sample size are needed to support future results.

## 1. Introduction

Human immunodeficiency virus (HIV) and malaria parasite interact synergistically, leading to worse health outcomes [1]. Frequent episodes of symptomatic malaria can lead to poorer health outcomes, including anemia, an increase in plasma viral load, and a drop in CD4 count in HIV-infected individuals [2–4]. HIV and malaria exhibit similar geographical distributions, predominantly affecting individuals in Sub-Saharan Africa, the Indian subcontinent, and Southeast Asia. Because of the overlap in their geographic distribution and the resulting rates of coinfection, interactions between the two diseases present significant public health concerns [5, 6]. In Ethiopia, malaria is thought to be prevalent in more than 75% of the country's territory, affecting over half of its population. The major plasmodium species in the country are *P. falciparum* and *P. vivax*, accounting for 60% and 40% of all cases, respectively [7, 8].

HIV/acquired immunodeficiency syndrome (AIDS) is the other debilitating disease that emerged after zoonotic infections with simian immunodeficiency viruses from African primates were transmitted from apes to human beings. C-C chemokine receptor type 5 (CCR5, also known as R5) and C-X-C chemokine receptor type 4 (CXCR4, also known as X4) are coreceptors found on human immune cells that are utilized by HIV strains to enter these cells. Both R5 and X4 HIV strains play an important role in disease pathogenesis, especially with the persistence of R5 HIV-1 strains at the AIDS stage. This shows that both R5 and X4 strains equally diminish cluster of differentiation 4 positive (CD4+) T cells and play their part in disease outcomes [9].

Malaria and HIV/AIDS coinfections have been suggested as the two most serious health challenges of developing countries, causing nearly 4 million deaths worldwide each year [10]. According to the study conducted in Sub-Saharan Africa, the prevalence of malaria-HIV/AIDS coinfection was 0.7%–47.5% in nonpregnant adults, 1.2%–27.8% in children, and 0.94%–37% in pregnant women. Malaria-HIV/AIDS coinfection was related with an increased number of clinical parasitemia and severe malaria, increased parasite and viral load, and reduced malaria immunity in pregnant adults [11]. In areas where malaria is highly prevalent, people with HIV/AIDS infection are prone to coinfection with malaria, particularly those who are immune-compromised [12]. Treatments against malaria more commonly fail in HIV-positive individuals with lower CD4-cell counts and anemic patients compared with those who are HIV-negative [13]. HIV also worsens the effects of malaria, enhances malaria communicability, and causes strong CD4 cell activation and expression of cytokines. HIV infection weakens the immune system, creating a condition that increases susceptibility to malaria, leading to higher parasite densities, more frequent and severe malarial episodes, and greater risk of adverse outcomes such as severe anemia and increased malaria transmission in malaria coinfected individuals. This interplay accelerates malaria progression and worsens disease severity [2].

In addition, HIV/AIDS patients are highly prone to critical malaria diseases. In conditions where there is concurrent infection with both diseases, HIV also increases the rate of antimalaria treatment failure. Malaria causes short-term elevations in viral load, worsening clinical disease and increasing feto-maternal transmission and transmission in adults. HIV-positive individuals of all age groups have higher chances of developing a critical malaria illness that leads to increased fatality rates because HIV causes a defective immune response [11, 14].

Both diseases can alter hematological profiles separately, in addition to other nonhematological problems. These hematological alterations increase the severity of malaria diseases and are one of the most commonly encountered health problems in malaria patients. The alterations include almost all cell lines [15]. The alterations involve the depletion of erythrocytes, thrombocytes, total leukocytes, increased lymphocytes, and others, even though malaria infection varies depending on different factors [16–18]. In individuals who are coinfected with HIV and malaria, changes in hematological parameters are the primary contributors to mortality when contrasted with those who have HIV alone, and both HIV and malaria are also diseases of poverty [19]. Moreover, HIV infection highly increases the development of severe malaria and leads to a more than 6 times increased chance of mortality [20].

In Ethiopia, coinfection is common, and the prevalence of anemia, thrombocytopenia, and leukopenia in malaria and HIV coinfected participants was 60.5%, 59.3%, and 43.0%, respectively [21].

However, there is limited information on the collective impact of malaria-HIV coinfection on hemoglobin levels, red blood cells (RBCs), white blood cells (WBCs), and other hematological profiles [3, 22, 23]. In addition, there are no reported data around the study area on the selected hematological profiles of HIV-malaria parasites coinfected adult individuals. Therefore, this study was designed to determine hematological profiles of HIV-malaria parasites coinfected adult individuals on highly active antiretroviral therapy (HAART) in Bonga Gebretsadik Shawo General Hospital, Southwest Ethiopia, 2022.

## 2. Materials and Methods

### 2.1. Study Area and Period

This study was conducted in Bonga town, Gebretsadik Shawo General Hospital, Southwest Ethiopia. There were more than 1000 registered individuals on regular HAART follow-ups in Bonga Gebretsadik Shawo General Hospital. This study was carried out from June 13 to November 3, 2022.

### 2.2. Study Design

A hospital-based comparative cross-sectional study was conducted.

### 2.3. Population

#### 2.3.1. Source and Study Population

The source population consisted of adult HIV/AIDS patients attending the Antiretroviral Therapy (ART) clinic at Bonga Gebretsadik Shawo General Hospital during the study period. The study population specifically included adult HIV/AIDS patients undergoing follow-up on HAART at the same hospital.

### 2.4. Sample Size and Sampling Technique

The sample size was determined using a statistical formula for comparing two population means, based on data from a previously conducted study at Gambella Hospital, Ethiopia [21]. Those study participants who were willing to participate in the study were included consecutively. Accordingly, a total of 196 HIV-positive patients (98 HIV infected and 98 HIV-malaria coinfected) were included in the study.

### 2.5. Eligibility Criteria

HIV-positive individuals whose age is 18 years or older coming to the ART clinic for regular HAART follow-up, patients who were not using antimalarial drugs for the past 2 months, and those willing to participate during the study period were included.

### 2.6. Data Collection and Processing

Structured questionnaires were used to collect sociodemographic data of the study participants. Five milliliters venous blood sample was collected by using a vacutainer tube from each study participant for laboratory investigation. An estimated 6 μL of blood was taken to prepare thick blood films from ethylenediaminetetraacetic acid (EDTA) anticoagulated venous blood and stained using 10% Giemsa solution for parasite detection and parasite density determination. Malaria parasite density was estimated from thick blood films by counting asexual parasites against 200 white blood cells, and subsequently calculated using the standard formula, assuming a reference white blood cell count of 8000 cells/μL.

The remaining blood sample was used for complete blood count (CBC) analysis and viral load. A complete blood count was performed using the Siemens (ADVIA 560, UK) machine, and viral load testing was done at Jimma University Medical Center using the Abbott RealTime HIV 1 assay. All the data collection processes were done by the principal investigator, ART focal person, and other senior laboratory technologists, and the results were recorded. Anemia is defined as a hemoglobin value below 12 g/dL for adult nonpregnant women and below 13 g/dL for adult men [24]. Leukopenia is defined as a number of total WBCs below 4 × 10^9^/L [25]. Thrombocytopenia is defined as platelet count lower than 150,000/μL [23].

To get reliable data, training was given to data collectors by the principal investigator. The standard operating procedure was followed for each test to ensure the quality of laboratory data. The reagent expiry date was checked before running the tests as per their manufacturer's instructions. Malaria slides were checked by two experienced laboratory technologists. Quality control procedures were performed daily according to laboratory protocols. Quality control samples were used for the CBC hematology analyzer and viral load count machine.

### 2.7. Data Processing and Analysis

Data generated were entered into EpiData 3.1, reviewed, cleaned, and exported into the Statistical Package for the Social Sciences (SPSS) software Version 25 for analysis. Descriptive analysis was used to see the distribution of the study participants' sociodemographic data and was displayed using tables. An independent sample *t*-test was performed to compare the mean differences in hematological profiles between the HIV-infected and HIV-malaria coinfected study participants. Correlation between hematological profiles and viral load, as well as between hematological profiles and parasite density was performed, and *p* < 0.05 was taken as a significance level.

## 3. Results

### 3.1. General Characteristics of the Study Population

Out of the total of 196 study participants, the majority, 139 (70.9%), were females with a mean age of 32.79 (±10.7) years, ranging from 18 to 63 years. Most of the study participants, 154 (78.6%), were Bonga town dwellers. All of the participants enrolled in this study were on HAART follow-up, and more than 95% had at least a primary education, with the rest being illiterate (4.6%). More than half of the study population, 103 (52.6%), were married. Regarding the occupation, the majority of the study participants were housewives, 52 (26.5%). Out of the total study participants, 164 (83.7%) drink coffee/tea immediately after a meal, and 191 (97.4%) of the study participants eat meat and dairy products, while the rest, 5 (2.6%), do not. Out of the total of 196 study participants, 133 (67.9%) have previously experienced malaria infection, while the remaining, 63 (32.1%), have not experienced any malaria infection (Table 1).

### 3.2. Hematological Profiles Among HIV-Infected and HIV-Malaria Coinfected Study Participants

A statistically significant difference was observed in the mean ± SD values of RBCs, Hgb, and HCT (*p* < 0.05) between HIV-infected and HIV-malaria coinfected study participants. A statistically significant difference was not observed in mean values of total WBC, neutrophils, lymphocytes, monocytes, MCV, MCHC, basophils, and platelets in both groups (*p* > 0.05) (Table 2).

### 3.3. Correlation of the Hematological Profile to Viral Load and Parasite Density

The malaria parasite density ranges from 40 to 24,000, with a median of 1020 parasites/μL. Significant negative correlation was observed between viral load and several hematological parameters, including total white blood cells, neutrophils, lymphocytes, eosinophils, red blood cells, hemoglobin, hematocrit, mean cell volume, and platelets. In the HIV-malaria coinfection, parasite density showed a negative correlation with red blood cells, hemoglobin, hematocrit, and platelets (Table 3).

### 3.4. Prevalence of Hematological Abnormalities in Both Groups

**Figure 1:**
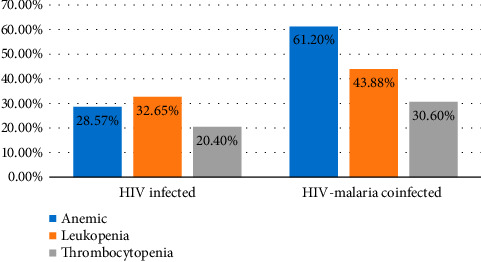
Prevalence of hematological abnormality in HIV-positive individuals and HIV-malaria positive individuals on HAART follow-up at ART Clinic in BGTSG, Southwest Ethiopia, from June 13 to November 3, 2022.

From a total of 196 participants, 88 (44.9%) had anemia, 77 (39.3%) had leukopenia, and 50 (25.5%) had thrombocytopenia. The prevalence of anemia, leukopenia, and thrombocytopenia among malaria-HIV coinfected study participants was 60 (61.2%), 43 (43.88%), and 30 (30.6%), respectively. A statistically significant difference was observed in the prevalence of anemia (*p* < 0.001) between the two groups (Figure 1). As the altitude of the study area is ∼1500 m above sea level, we adjusted the hemoglobin value by subtracting 0.5 g/dL from each range [24].

## 4. Discussion

This study has attempted to provide information on the hematological profiles of HIV-infected and HIV-malaria coinfected study participants at Bonga Gebretsadik Shawo General Hospital. The major findings are as follows. The prevalence of anemia, leukopenia, and thrombocytopenia among malaria-HIV coinfected study participants was higher than malaria noninfected participants. The mean hematological profiles were lower in study participants with malaria coinfection. Hematological profiles were correlated with viral load in both groups and parasite density in malaria coinfection.

Malaria-HIV coinfection is found to have a greater effect in reducing the mean value of RBCs, Hgb, and HCT as compared to only HIV-infected participants. This finding is consistent with the findings of other studies, like the studies conducted in Gambella [21], Nigeria [26], USA [22], and Ghana [3]. However, this study contradicts with study done in Cameroon [27]. The difference might be due to the small number of study participants involved in the Cameroon study. However, in our study, there was no significant difference in the mean value of MCV, MCH, and MCHC in malaria-infected and nonmalaria-infected HIV participants. This is also supported by a study done in Cameroon [27]. This might be due to the normocytic normochromic nature of anemia in malaria and HIV infections.

The reduction in RBCs, Hgb, and HCT in coinfected individuals as compared to the HIV-infected ones might be due to increased destruction of infected red cells (both old and young in the case of *Plasmodium falciparum*) and decreased production of red cells due to dyserythropoiesis. The other possible mechanism is marked splenomegaly during acute infection, reflecting extensive sequestration of red cells by the spleen, resulting in anemia [28].

This study showed that most hematological parameters (WBCs, neutrophils, lymphocytes, eosinophils, RBCs, hemoglobin, hematocrit, mean cell volume, and platelets) were negatively correlated with viral load. This finding agrees with studies conducted in India [29] and Ethiopia [30]. This might be because the level and change in viral load are important indicators of HIV disease progression [31]. This might affect processes that are important during the early stages of hematopoiesis or stem cell differentiation, leading to a spectrum of morphological changes within the bone marrow microenvironment that are strongly associated with various forms and levels of peripheral blood pancytopenia [32].

In individuals with HIV-malaria coinfection, parasite density was negatively correlated with red blood cell count, hemoglobin level, hematocrit, and platelet count. This finding agrees with the findings of studies conducted in Nigeria in 2012 [33] and 2022 [10] and Indonesia [34]. The possible causes for this might be due to the removal of red cells containing parasites from the circulation by the reticuloendothelial system. There is also accelerated destruction of nonparasitized cells and dyserythropoiesis in the bone marrow. Parasitized and nonparasitized red cells lose deformability, and the high shear rates in the spleen enhance their removal by that organ. This might be worsened by parasite density, although parasite density does not necessarily correlate with disease severity [35].

In this study, the prevalence of anemia was higher, 60 (61.2%), in HIV-malaria coinfected groups than in malaria-negative HIV-infected participants, 28 (28.57%). This finding was in line with another study [21]. However, the prevalence of anemia in HIV malaria coinfected was lower as compared to studies done in Ghana [36], Nigeria [37], and Ethiopia [38], which reported 97.1%, 66.7%, and 71.3%, respectively. However, our study findings were higher than the study findings conducted in Abuja (4.8%) [39]. This difference might be due to methods like sample size variation, and also, the study's use of clinical conditions like the HAART status of the study participants. Anemia in malaria might be attributed to direct lysis of infected RBCs during schizogony, immune destruction of infected and noninfected RBCs in the spleen, and inhibition of erythropoiesis and ineffective erythropoiesis [40].

Leukopenia was identified as another hematological abnormality, affecting 43 (43.88%) of individuals in the HIV-malaria coinfected group and 32 (32.65%) of those with HIV infection alone. This report is lower than the study done in Nigeria, which is 63.3% in HIV-malaria coinfected groups [37]. The difference might be due to variations in the cutoff value used for the leukopenia. In Nigeria, they used a leukopenia cutoff value of ≤ 3 × 10^9^/L, but in our study, we used a leukopenia below 4 × 10^9^/L. The reduction in leukocyte count might be due to the localization of leukocytes away from the peripheral circulation rather than actual depletion or stasis [41].

Among HIV-malaria coinfected groups, 30 (30.6%) had thrombocytopenia, and among malaria-negative HIV-infected participants, 20 (20.4%) had thrombocytopenia. This finding is lower than the study conducted in Nigeria (60%) and Ethiopia (59.3%) [21, 37]. In malaria, both immune and nonimmune factors have been recognized as contributors to thrombocytopenia due to their roles in platelet destruction. The proposed mechanisms include disturbances in coagulation, sequestration within the spleen, destruction of platelets due to antibodies, oxidative stress, and the involvement of platelets as cofactors in the onset of severe malaria. Changes in the structure and function of platelets have been noted as a result of malaria, and in rare cases, malaria parasites can directly invade platelets [42–44]. The limitations of the study are as follows. The study did not address associated risk factors, failed to identify species of malaria, included small sample size which may limit generalizability, and included the study participants who were on HAART only.

## 5. Conclusions

Our findings showed that the mean values of hematological profiles are significantly lower in HIV-malaria coinfected participants than in the HIV-infected participants. The study also revealed that there was a relatively strong negative correlation between the values of most of the hematological profiles and viral load in both groups. There was also a strong negative correlation between the values of most of the hematological profiles and parasite density in HIV-malaria coinfected study participants. The study further indicated that the prevalence of anemia is higher among malaria-HIV coinfected study participants than among study participants infected with HIV alone. To strengthen future research, we recommend incorporating comprehensive parasite species identification and studying cause-and-effect relationships with large sample sizes.

## Figures and Tables

**Table 1:** General characteristics of study participants, Bonga Gebretsadik Shawo General Hospital, Southwest Ethiopia, from June 13 to Nov 3, 2022 (*n* = 196).

Variable	Frequency	Percent
Gender		
Male	57	29.1
Female	139	70.9
Age		
18–29	82	41.8
30–39	66	33.8
40–49	26	13.2
≥ 50	22	11.2
Residence		
Urban	154	78.6
Rural	42	21.4
Occupation		
Farmer	21	10.7
Housewife	52	26.5
Merchant	29	14.8
Government employee	47	24
Student	44	22.4
Driver	3	1.5
Educational status		
Illiterate	9	4.6
1–8	68	34.7
9-10	39	19.9
11-12	25	12.8
College/university	55	28.1
Marital status		
Married	103	52.6
Single	75	38.3
Widowed	13	6.6
Divorced	5	2.6
Take tea/coffee immediately after a meal		
Yes	164	83.7
No	32	16.3
Eat fruit and vegetables		
Yes	196	100
No	—	—
Eat meat and dairy products		
Yes	191	97.4
No	5	2.6
Previous malaria experience		
Yes	133	67.9
No	63	32.1

**Table 2:** Comparison of hematological profiles between HIV-infected and HIV-malaria coinfected study participants, Bonga Gebretsadik Shawo General Hospital, Southwest Ethiopia, from June 13 to Nov 3, 2022 (*n* = 196).

	Participant group	Mean ± SD	*t*-test	*p* value
WBC	HIV-malaria coinfected	4.54 ± 1.49	−0.785	0.43
	HIV infected	4.70 ± 1.42		

Neutrophil	HIV-malaria coinfected	2.28 ± 1.10	−1.136	0.26
	HIV infected	2.45 ± 1.04		

Lymphocyte	HIV-malaria coinfected	1.50 ± 0.630	−0.105	0.92
	HIV infected	1.51 ± 0.72		

Monocyte	HIV-malaria coinfected	0.41 ± 0.51	−0.139	0.89
	HIV infected	0.42 ± 0.52		

Eosinophil	HIV-malaria coinfected	0.30 ± 0.46	0.801	0.42
	HIV infected	0.24 ± 0.43		

Basophil	HIV-malaria coinfected	0.03 ± 0.17	1.750	0.082
	HIV infected	0.00 ± 0.00		

RBC	HIV-malaria coinfected	3.69 ± 1.06	−5.202	< 0.001
	HIV infected	4.44 ± 0.94		

Hgb	HIV-malaria coinfected	10.52 ± 2.93	−5.616	< 0.001
	HIV infected	12.65 ± 2.35		

HCT	HIV-malaria coinfected	30.08 ± 8.32	−5.412	< 0.001
	HIV infected	35.88 ± 6.58		

MCV	HIV-malaria coinfected	77.52 ± 7.12	−1.287	0.2
	HIV infected	78.63 ± 4.74		

MCH	HIV-malaria coinfected	27.42 ± 4.45	0.63	0.53
	HIV infected	27.10 ± 2.21		

MCHC	HIV-malaria coinfected	34.49 ± 3.73	−1.467	0.14
	HIV infected	35.36 ± 4.51		

Plt	HIV-malaria coinfected	221.7 ± 119.995	−0.689	0.49
	HIV infected	232.13 ± 89.73		

*Note:* Hgb, hemoglobin; HCT, hematocrit; Plt = platelet.

Abbreviations: MCH, mean corpuscular hemoglobin; MCHC, mean cell hemoglobin concentration; MCV, mean cell volume; RBC, red blood cell; WBC, white blood cell.

**Table 3:** Correlation of the hematological profile with viral load and parasite density, Bonga Gebretsadik Shawo General Hospital, Southwest Ethiopia, from June 13 to Nov 3, 2022 (*n* = 196).

Hematological profiles	Viral load in both groups		Parasite density in HIV-malaria coinfected study participants	
	Correlation coefficient	*p* value	Correlation coefficient	*p* value
TWBCs (× 10^3^/μL)	−0.53	0.0001	−0.193	0.0574
Neutrophil (× 10^3^/μL)	−0.48	0.0001	−0.19	0.0650
Lymphocyte (× 10^3^/μL)	−0.41	0.0001	−0.164	0.1072
Monocyte (× 10^3^/μL)	−0.13	0.0626	−0.057	0.5726
Eosinophil (× 10^3^/μL)	−0.15	0.0407	0.076	0.4585
Basophil (× 10^3^/μL)	−0.11	0.1209	−0.075	0.4625
RBCs (× 10^6^/μL)	−0.39	0.0001	−0.702	0.0001
Hgb (g/dL)	−0.46	0.0001	−0.77	0.0001
HCT (%)	−0.45	0.0001	−0.796	0.0001
MCV (fl)	−0.15	0.0309	−0.16	0.1170
MCH (pg)	0.015	0.8331	0.197	0.0512
MCHC (%)	−0.021	0.7726	0.096	0.3454
Plt (× 10^3^/μL)	−0.29	0.0001	−0.56	0.0001

## Data Availability

The data that support the findings of this study are available from the corresponding author upon reasonable request.
